# Older adults are impaired in the release of grip force during a force tracking task

**DOI:** 10.1007/s00221-023-06770-y

**Published:** 2024-01-22

**Authors:** Sara Davidson, Kenneth Learman, Eric Zimmerman, Anson B. Rosenfeldt, Mandy Koop, Jay L. Alberts

**Affiliations:** 1 Department of Biomedical Engineering, Cleveland Clinic, 9500 Euclid Avenue, ND-20, Cleveland, OH 44195 USA; 2 Youngstown State University, Youngstown, OH USA; 3 Cleveland Clinic, Center for Neurologic Restoration, Cleveland, OH USA

**Keywords:** Aging, Grip force, Force modulation, Dexterous function, Force release

## Abstract

Age-related changes in force generation have been implicated in declines in older adult manual dexterity. While force generation is a critical aspect of the successful manipulation of objects, the controlled release of force represents the final component of dexterous activities. The impact of advancing age on the release of grip force has received relatively little investigation despite its importance in dexterity. The primary aim of this project was to determine the effects of age on the control of force release during a precision grip tracking task. Young adults (*N* = 10, 18–28 years) and older adults (*N* = 10, 57–77 years) completed a ramp-hold-release (0–35% of maximum grip force) force tracking task with their dominant hand. Compared to young adults, older adults were disproportionately less accurate (i.e., less time within target range) and had more error (i.e., greater relative root mean squared error) in the release of force, compared to generation of grip force. There was a significant difference between groups in two-point discrimination of the thumb, which was moderately correlated to force control across all phases of the task. The decline in force release performance associated with advanced age may be a result of sensory deficits and changes in central nervous system circuitry.

## Introduction

Declines in fine motor control and manual dexterity begin in middle age and worsen after 60 years of age (Cole et al. 1999; Dayanidhi and Valero-Cuevas 2014; Hackel et al. 1992; Lindberg et al. 2009; Smith et al. 1999). Several physiological changes have been linked to declines of dexterous function associated with healthy aging. Most common contributors include: muscle atrophy, changes in the number of available motor units and the speed of their recruitment, decreased tactile acuity due to cutaneous mechanoreceptor degradation, and reduced fingertip friction due to increased skin slipperiness associated with aging (Carmeli et al. 2003; Cole et al. 1999; Gorniak and Alberts 2013). Understanding specific aspects of fine motor control and hand function affected by the aging process will aide in understanding aging and provide insight into potential approaches to prevent or slow these dexterous declines that contribute to a loss of independence in older adults (Incel et al. 2009; Ostwald et al. 1989; Rattanawan 2022).

The objective quantification of hand function has evolved from timed motor tasks such as placing pegs in holes (Sterne 1969), to kinematic measurements while reaching to and grasping an object (Jeannerod 1984) and the use of force transducers to characterize the kinetic aspects of object manipulation (Cole 1991; Cole et al. 1999; Westling and Johansson 1984). Generally, analysis of force control in older adults indicates deficits in smooth and controlled force generation and maintenance of submaximal forces (Francis et al. 2012; Galganski et al. 1993; Kurillo et al. 2004; Lindberg et al. 2009; Vaillancourt et al. 2002; Voelcker-Rehage and Alberts 2005) which likely underlies a strategy of over gripping objects (Cole 1991; Cole et al. 1999; Gorniak and Alberts 2013; Kinoshita and Francis 1996).

The changes in force generation and maintenance in older adults are relatively well defined (Galganski et al. 1993; Lindberg et al. 2009; Ranganathan et al. 2001; Spirduso and Choi 1993; Vaillancourt et al. 2003; Voelcker-Rehage et al. 2006). However, the impact of advanced age on the control and coordination of the release of force has received little attention. As a requisite for the manipulation and handling of objects, force release is a vital “final component” of skilled dexterous function. Efficient performance of activities of daily living, such as tying laces, buttoning a shirt, and object manipulation and movement, requires the efficient generation and release of digit forces.

Dysregulation of grip forces has undesirable effects such as fatiguing hand muscles or damaging fragile objects, and inaccurate object placement. Among young adults, force accuracy and variability are worse during index finger flexion force release than force generation (Patel et al. 2019), suggesting that force generation and release are distinct tasks that rely on different neural networks. Compared to force generation, functional magnetic resonance imaging during unimanual force release in young adults demonstrated decreased activity in the contralateral primary motor cortex and bilateral caudate nucleus, increased activity in the right dorsolateral prefrontal cortex, and increased deactivation in the anterior cingulate cortex (Spraker et al. 2009). This provides evidence that force release is modulated by a neural process that may be distinct from force generation and should be evaluated as a potential contributor to age-related changes in manual dexterity.

Force release among older adults is less studied and there are conflicting reports regarding the impact of aging on force release. Some demonstrate little impact of aging (Lindberg et al. 2009; Naik et al. 2011), while others have demonstrated that older adults have more difficulty with controlled grip force release during a sine tracking task (Voelcker-Rehage and Alberts 2005) and that switching from force generation to release is more difficult for older adults than younger adults (Francis et al. 2012). The limited amount of understanding specifically examining the effects of age on grip force release represents a gap in knowledge of the effects of aging on motor control.

The primary aim of this project was to determine the effects of age on pattern of force release during a force tracking task with distinct force generation, maintenance, and release phases. The secondary aim was to determine if age had a greater impact on grip release compared to grip generation or maintenance. It was hypothesized that older adults would exhibit greater variability during force release compared to force generation and maintenance phases.

## Methods

### Participants

Using similar force tracking projects as a guide (Naik et al. 2011; Voelcker-Rehage and Alberts 2005), data from 24 young and older adults were collected. Four participants were removed from analysis due to equipment calibration error (*n* = 2), essential tremor (*n* = 1) and pre-existing dominant hand injury (*n* = 1). A total of 20 participants were included in the analysis: 10 young healthy adults and 10 older adults (Table 1 includes demographics). All participants were neurologically healthy with normal hearing and vision. The study was approved by the Cleveland Clinic Institutional Review Board and all participants underwent the informed consent process in accordance with the Declaration of Helsinki.

**Table 1:** Participant demographics, presented as mean ± standard deviation for continuous variables, median [IQR] for ordinal and interval variables, and *N* (%) for categorical variables

	Young adults (*N* = 10)	Older adults (*N* = 10)
Age (years)	22.6 ± 2.8	67.7 ± 7.2
Male sex, *n*	4 (40%)	4 (40%)
Race, *n*		
African American	1 (10%)	1 (10%)
White	9 (90%)	9 (90%)
Hispanic ethnicity, *n*	0 (0%)	0 (0%)
Dominant side right (versus left), *n*	7 (70%)	10 (100%)
SWMT (dominant side)		
Thumb	2.83 [2.83, 2.83]	2.83 [2.83, 3.61]
Index	2.83 [2.83, 2.83]	2.83 [2.83, 2.83]
TPD (dominant side)		
Thumb (mm)	2.50 [2.00, 3.00]	4.00 [3.25, 4.00]
Index (mm)	3.00 [2.00, 3.00]	3.50 [2.25, 4.00]

*SWMT* Semmes–Weinstein Monofilament Testing, *TPD* two-point discrimination

### Instrumentation

Grip force data were collected using a Mini-40 force-torque transducer (ATI Industrial Automation, Garner, NC, USA) encased in a custom aluminum housing. Sampling rate was 100 Hz for max grip force collection and 30 Hz for tracking. Visual display of the task, composed of a ramp up-static hold-ramp down time (described in detail subsequently), was provided on a computer monitor positioned in front of the participant. Using a custom Python 3 script, real-time performance data from the participant were displayed on a computer monitor to provide immediate knowledge of performance.

### Participant positioning

Participants sat in a straight-backed armless chair in front of a table, on which the force transducer and computer monitor were situated at midline. Participants’ elbows were flexed approximately 90 degrees when their hands were resting on the table edge.

### Assessments

#### Maximum grip force

Maximum voluntary contraction (MVC) of dominant hand precision grip was collected across three trials with a 2 min rest break between trials. The maximum force value (N) of those trials was used as the participant’s maximum and used to create participant-specific force tracking values.

#### Sensory testing

Sensory testing was performed on the dominant thumb and index finger. Semmes–Weinstein Monofilament Testing (SWMT) and two-point discrimination (TPD) were performed per standard procedures (Bell-Krotoski et al. 1993; Dellon et al. 1987). Briefly, the SWMT quantifies the light touch threshold by touching the pad of the digit with monofilaments ranging in size from 2.83 to 6.65, with lower values indicating that the monofilament requires less force to bend it. The thinnest monofilament that the individual can accurately sense with their eyes closed is recorded. Two-point discrimination is a test of sensory nerve density where the examiner touches the pad of the digit with either one or two points. With their eyes closed, individuals state how many points they feel. If the participant can accurately identify the two-point stimulus, the distance between the two points is decreased until they are no longer able to discern two points. The smallest distance (mm) between two points that the subject can accurately identify two out of three trials is recorded as the limen.

#### Force tracking

**Fig. 1:**
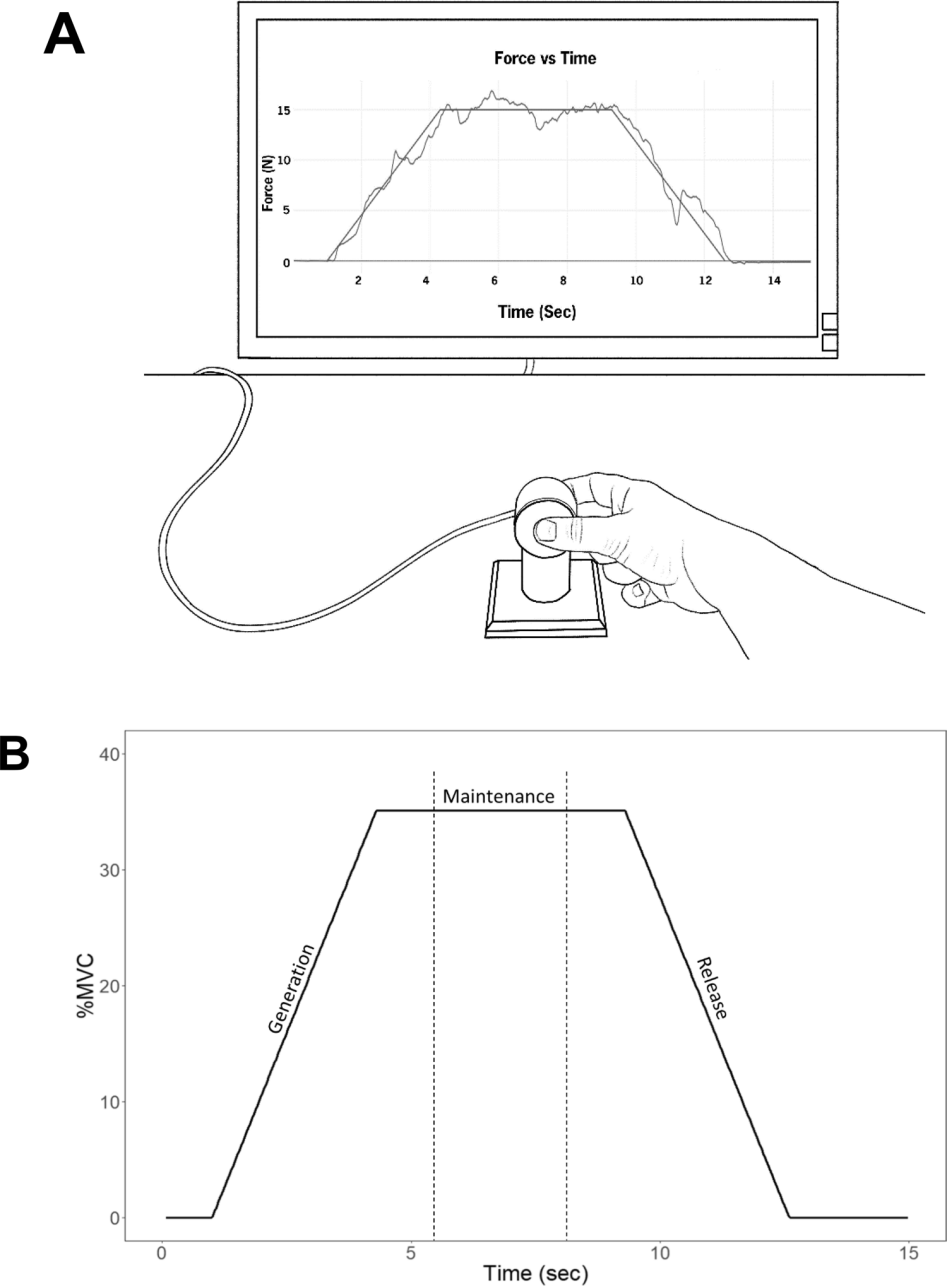
**A** Illustration of the force transducer, computer monitor providing visual feedback and participant using a precision grip to complete the force tracking task. **B** The target force for the tracking trials. Based on the slope of the target force, tracking data were separated into generation, maintenance, and release phases for analysis. To capture a steady state, the first and the last second of the maintenance phase were removed from analysis, indicated by the vertical dashed lines. *MVC* maximum voluntary contraction

The force tracking task recorded the force (N) generated during the task while using precision grip of the dominant hand. The force tracking task consisted of three phases: force generation that required increasing force production (0–35% of MVC), followed by a five second force maintenance period at 35% of MVC, and concluding with a force release phase from 35 to 0% MVC. The force generation and release phases were each 3.3 s in length (reflecting an approximate rate of force generation of 10% MVC/s) (Naik et al. 2011). A one second period at 0% MVC was provided before force generation to allow time for hand placement. Real-time feedback of grip force produced was superimposed over the target force pattern on the computer screen, and participants were instructed to trace the target force as accurately as possible. See Fig. 1 for an example of the experimental setup and the force tracking trial. Following 1–2 familiarization trials to ensure task understanding, 10 trials were collected.

#### Fatigue

Ratings of hand fatigue were recorded after the force tracking trials. A visual analog scale from 0 to 100 was provided, with 100 indicating total fatigue.

### Data reduction

Raw force tracking data were analyzed offline using custom MATLAB (R2021a) scripts. Linear interpolation was applied to the raw force tracking data to ensure uniform samples of 30 Hz. The resampled data were filtered with a 2nd-order Butterworth filter (12 Hz cutoff). Force tracking trials were separated into generation, maintenance, and release phases based on the slope of the target force (see labels in Fig. 1). The entire 3.3 s of generation and release phases were included in analysis. The middle 3 s of the 5 s “hold” phase was included for the maintenance phase analysis to best reflect steady state performance. Relative Root Mean Squared Error (RRMSE) and Percent Time Within 5% of Target Range (%TWR) were calculated for each phase.

#### Relative root mean squared error

To quantify the amount of error during the force tracking, the RRMSE was calculated using formula 1. This method quantifies the magnitude of the error relative to grip force values, where *F*_T_(*t*) is the target force, *F*_0_(*t*) is the actual force produced, and *T* is the length (sec) of the phase. A lower RRMSE represents decreased error with force tracking.

1 $${\text{RRMSE}}\, = \,\sqrt {\frac{1}{T}\mathop \sum \limits_{t = 0}^{T} \frac{{\left( {F_{0} \left( t \right) - F_{{\text{T}}} \left( t \right)} \right)^{2} }}{{\max \left( {F_{{\text{T}}} } \right)^{2} }}} .$$

#### Percent time within 5% of target range

Accuracy was calculated to determine percent of time spent ± 5% of the target force. A higher %TWR reflects greater task accuracy. Unlike RRMSE, %TWR quantifies how consistently the participant remains in close proximity to the target force.

### Statistical analysis

Statistical analyses were completed using R software (version 4.2.2). Normality was confirmed via Q–Q plots of the residuals and with Shapiro–Wilk tests. Effects of age on MVC, sensation, and fatigue (self-reports from 0 to 100 on a visual analog scale) were assessed with Welch’s *t* tests or Mann–Whitney *U* tests, depending on normality. To answer the primary question regarding the effects of age on grip release, Welch’s *t* tests were used to compare young versus older adult performance on release phase metrics. To address the secondary aim and determine if age had a greater impact on grip release compared to grip maintenance or generation, separate 2 × 3 (group × phase) repeated measures ANOVAs were performed for RRMSE and %TWR. Welch’s *t* tests for generation and release phase metrics were run as follow-up testing for interaction effects and to provide context. Effect sizes were calculated with Hedges’ *g*, with thresholds of 0.2 for small effects, 0.5 for medium effects, and 0.8 for large effects (Cohen 1992). Spearman’s correlation coefficients were calculated to assess relationships between sensory outcomes and force tracking performance.

## Results

### Maximum grip force

There were no significant differences between groups for MVC (*W* = 47, *p* = 0.85). Median max grip (N) was 57.68 [46.98, 61.39] for young adults and 58.77 [44.92, 61.55] for older adults.

### Fatigue

Level of fatigue was minimal for both groups, with older adults reporting less fatigue (18.1 ± 16.8) than young adults (31.5 ± 19.9). These differences were not significantly different between groups; Welch’s *t* test results of *t*(17.51) = 1.63, *p* = 0.12.

### Sensory testing

Median SWMT, provided in Table 1, was not significantly different between groups for either digit (thumb: *W* = 65, *p* = 0.14; index: *W* = 50, *p* = 1.00). Compared to young adults, median TPD was significantly worse in the older adult group in the thumb (*W* = 83, *p* = 0.009) but not the index finger (*W* = 67, *p* = 0.19).

### Force tracking

**Fig. 2:**
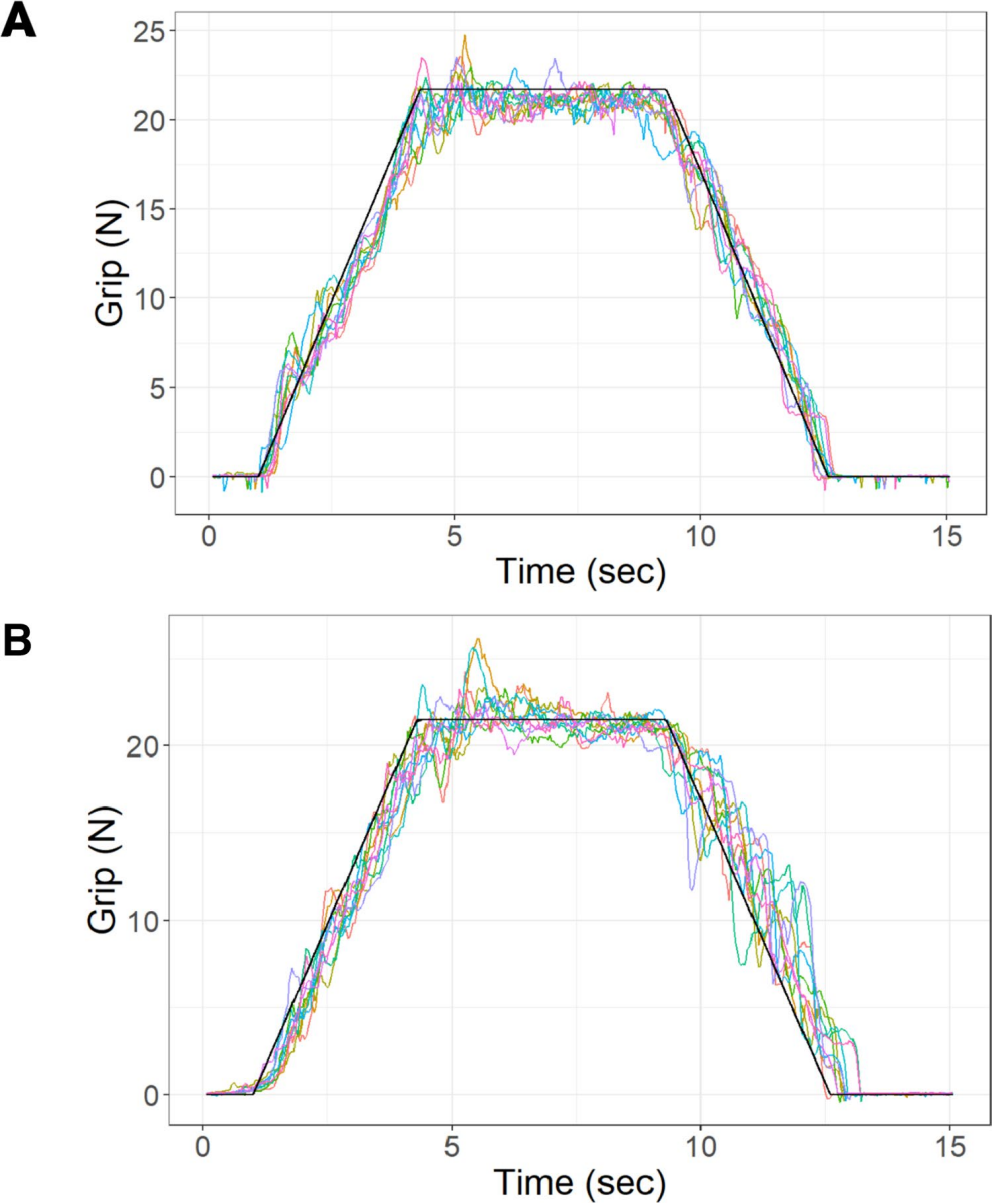
Representative tracking data from **A** a young adult and **B** an older adult. The straight black line indicates target force, with a maximum force of 35% of the participant’s maximum voluntary contraction. All ten tracking trials are overlaid on the target force

Examination of force traces (Fig. 2) and mean values (Table 2**)** suggests that young adults outperformed older adults on every variable. Repeated measures ANOVAs for both variables (see Table 2) confirmed significant differences between groups (RRMSE *F*_1,18_ = 11.84, *p* = 0.003; %TWR *F*_1,18_ = 11.97, *p* = 0.003) and phase (RRMSE *F*_2,36_ = 21.01, *p* < 0.0001; %TWR *F*_2,36_ = 24.11, *p* < 0.0001). Based on follow-up pairwise comparisons, both groups had less error and greater accuracy during the maintenance phase compared to the generation or release phases (all *p* < 0.0001).

**Table 2:** Means and standard deviations for each variable, by phase and group [relative root mean squared error (RRMSE), percent of time within 5% of target (%TWR)]

Variable	Generation		Maintenance		Release		Group *P* value	Phase *P* value	Interaction *P* value
	Young	Older	Young	Older	Young	Older			
RRMSE	0.47 ± 0.16	0.61 ± 0.20	0.24 ± 0.07	0.45 ± 0.31	**0.39** ± **0.06**	**0.75 ± 0.17**	.003	< .0001	.02
%TWR	**44.33 ± 14.08**	**31.28 ± 11.28**	75.36 ± 14.25	51.89 ± 32.43	**50.07 ± 6.45**	**28.07 ± 5.81**	.003	< .0001	.41

Bold text indicates a significant difference between groups based on pairwise comparisons

**Fig. 3:**
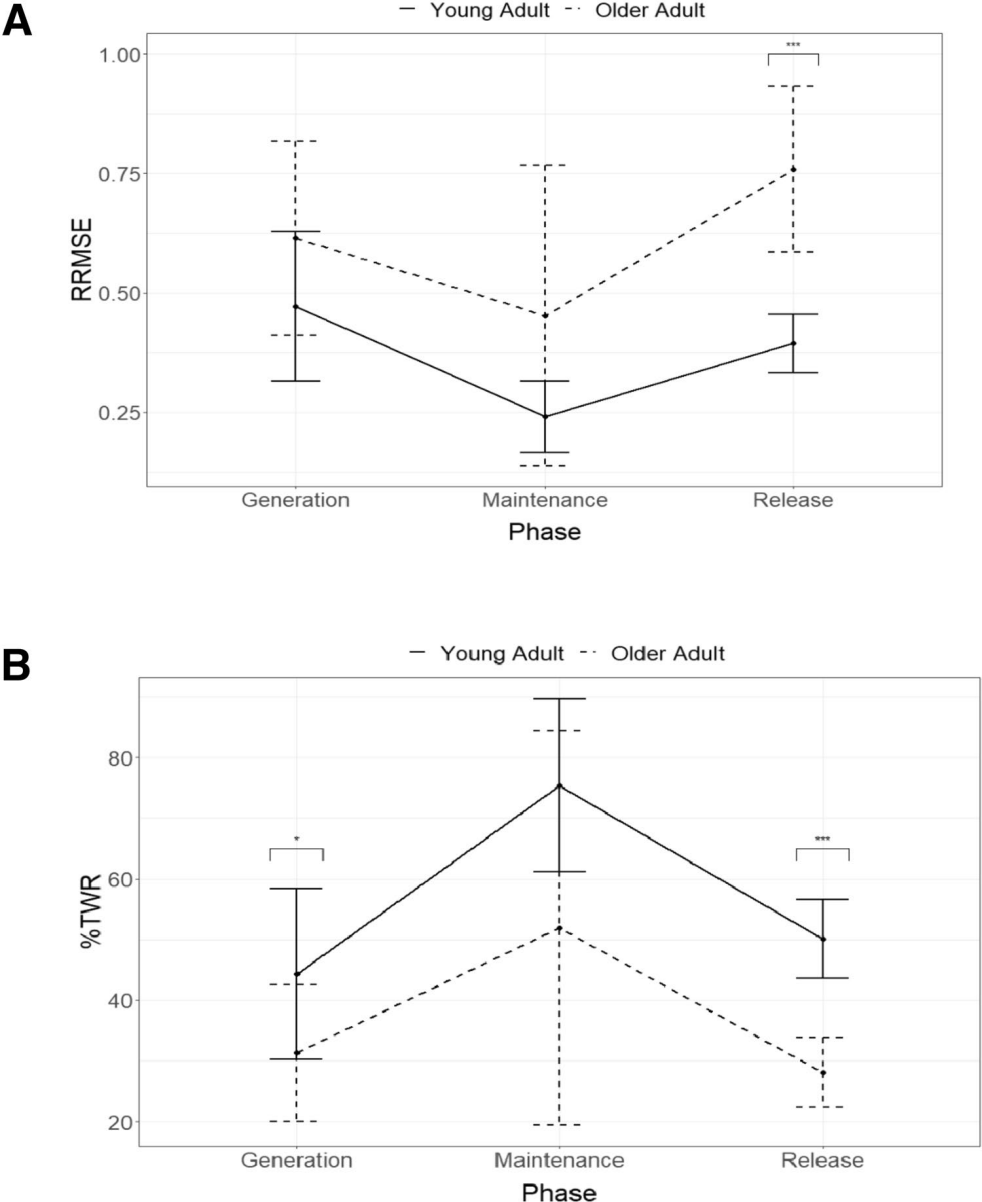
Comparisons of means and standard deviations for **A** relative root mean squared error (RRMSE) and **B** percent of time within 5% of target range (%TWR) during each phase, sorted by group. *p ≤ .05, ***p ≤ .001

There was a significant group × phase interaction for RRMSE (*F*_2,36_ = 4.37, *p* = 0.02) but not %TWR (*F*_2,36_ = 0.91, *p* = 0.41). As shown in Fig. 3, the interaction was driven by age-related changes in the release phase. Follow-up pairwise comparisons (Table 3) indicated that older adults performed significantly worse than young adults for both variables during force release (RRMSE *p* < 0.0001; %TWR *p* < 0.0001). Performance during the maintenance phase did not significantly differ between groups for either variable (RRMSE *p* = 0.07; %TWR *p* = 0.06). Older adults had a similar magnitude of error during force generation (RRMSE *p* = 0.09) but spent less time near the target range (%TWR *p* = 0.04). Detailed statistics, including effect sizes with 95% CI, are provided in Table 3, and Fig. 3 summarizes pairwise results.

**Table 3:** Results of pairwise comparisons between young and older adults

	*t*	*df*	*P*	Hedges’ *g* (95% CI)
Generation				
RRMSE	− 1.76	16.91	.10	0.75 (− 0.13, 1.62)
%TWR	2.29	17.18	.04	− 0.98 (− 1.87, 0.07)
Maintenance				
RRMSE	− 2.07	10.02	.07	0.89 (− 0.01, 1.76)
%TWR	2.09	12.35	.06	− 0.90 (− 1.77, 0.00)
Release				
RRMSE	− 6.27	11.20	< .001	2.69 (1.46, 3.88)
%TWR	8.01	17.81	< .0001	− 3.43 (− 4.81, -2.02)

### Sensory test correlations

Two-point discrimination of the thumb was moderately to strongly correlated with all tracking variables except force generation %TWR; TPD of the index was moderately to strongly correlated with all generation and maintenance tracking variables; SWMT of the thumb and index had no significant correlations with tracking variables. See Table 4 for correlation coefficients and significance.

**Table 4:** Correlations between sensory testing and force tracking performance

	TPD thumb	TPD Index	SWMT thumb	SWMT Index
Generation				
RRMSE	0.49*	0.43*	0.27	0.09
%TWR	− 0.52	− 0.48*	− 0.13	− 0.06
Maintenance				
RRMSE	0.48*	0.63**	− 0.01	− 0.17
%TWR	− 0.49*	− 0.70**	0.05	0.23
Release				
RRMSE	0.59**	0.22	0.13	0.00
%TWR	− 0.61**	− 0.28	− 0.26	0.06

*TPD* two-point discrimination, *SWMT* Semmes–Weinstein monofilament testing, *RRMSE* relative root mean squared error, %*TWR* percent of time within ± 5% of target range

*p ≤ .05, **p ≤ .01

## Discussion

There was a group × phase interaction effect for RRMSE, which was driven by very high values in the older adults during the release phase (see Fig. 3; Table 3). Examination of the representative force tracking trials and the pairwise comparison results suggest that while older adults are generally able to remain close to the target force during force generation and maintenance (reflected by similar RRMSE compared to young adults), they tend to have difficulty modulating the release of grip force in a controlled and coordinated manner compared to young adults, as evidenced by the high variability (see Figs. 2, 3). This change in the control of force release results in an increase in RRMSE observed in the older adult group and appears to be an ineffective compensation mechanism, given that older adults spent more time outside of the target range (increased %TWR) compared to young adults. Notably, differences in force release were not due to an increase in fatigue or difference in maximum grip force between the groups as fatigue and MVC did not differ between groups.

The accuracy and the precision of force control were impacted by the requirements of the task. The maintenance phase was the most stable of the three phases, suggesting that active control and modulation of grip force is more challenging than force maintenance for both young and older adults. The lack of difference between older and young adults during the maintenance phase is not surprising given that the effects of aging on constant force maintenance are most apparent at forces much lower than what was used in our study (Galganski et al. 1993; Vaillancourt et al. 2003).

The decreased accuracy (measured by %TWR) in both the generation and release phases among older adults suggests that age results in a general decline in the ability to precisely modulate grip force. Our findings confirm previous work demonstrating a global decline in grip force tracking performance with age (Kurillo et al. 2004; Spirduso and Choi 1993; Vieluf et al. 2013; Voelcker-Rehage and Alberts 2005), and expand upon a smaller body of evidence specifically examining grip release in older adults. Several studies have examined the effects of age on *immediate* grip force release, with one study finding no differences between young and older adults (Lindberg et al. 2009) and two others finding that older adults were slower to relax grip force (Lee et al. 2015, 2014). Studies examining the effect of age on *controlled* force release are scarce but have conflicting results. One study showed no difference between young and older adults (Naik et al. 2011), while another found that older adults were less accurate, even with practice (Voelcker-Rehage and Alberts 2005). There are several possible reasons for the group differences with controlled force release seen in our study, including sensory differences, fixed task speed, and changes in central nervous system circuitry.

Compared to young adults, older adults had poorer TPD of the dominant thumb, indicating a decline in the innervation density of slowly adapting large myelinated fibers (Dellon and Kallman 1983). Since TPD was correlated to most force tracking performance variables across all three phases of force tracking, group force tracking performance differences likely are related in part to age-related sensation degradation. Previous studies have found decline in performance with TPD and SWMT with aging (Kaneko et al. 2005; Logue et al. 2022). Although there were no group differences with SWMT, TPD has been found to correlate with object identification (Novak et al. 1993a, b) and predicts performance for tasks that require sustained pinch grip, more so than the SWMT (Dellon and Kallman 1983).

Reduced density of sensory fibers in the thumb of older adults could contribute to their increased RRMSE across phases, as it could indicate an increased reliance on visual feedback to correct deviations from the target as compensation for decreased sensation. The significant difference between groups for generation and release %TWR suggests that visual compensation for decreased sensation in older adults is an ineffective strategy. Given the robust effect sizes (see Table 3), this is especially true for the release phase.

It is possible that differences between groups were elicited because the speed of our task was fixed. A previous study found little to no difference between young and older adults among a force tracing task (Francis et al. 2012). Unlike force tracking, force tracing does not have a temporal requirement, and thus participants are able to perform the task at a self-selected speed. Future studies should examine differences between force tracking and force tracing among older adults.

Motor control strategies differ for force generation and release (Park et al. 2016; Spraker et al. 2009). Compared to simple motor tasks, more complicated motor tasks that required a combination of force generation and release showed more diffuse, bilateral activation patterns in the brains of young adults. Older adults demonstrate these diffuse, bilateral patterns with far simpler tasks, which is thought to be a strategy to compensate for age-related changes (Loibl et al. 2011; Ward and Frackowiak 2003; Ward et al. 2008) and likely is related to increased utilization of cognitive resources during motor tasks (Ward and Frackowiak 2003). The relationship between age and white matter volume follows an inverted U pattern across the lifespan, with both young children and older adults demonstrating the lowest volume of white matter (Leversen et al. 2012). Lower levels of white matter likely contribute to similarities in motor performance between older adults and young children (Leversen et al. 2012). Hence, the mechanisms behind decline in force release in old age may mirror the development of force release in childhood, and further investigation into similarities between the development of force control in children and the decline of force control in older adults may provide further insights into aging.

Additionally, with age, there is a reduction in the size and discharge rate of motor units (Park et al. 2016; Piasecki et al. 2016). Reduced motor unit size and discharge rates, coupled with age-related changes in agonist motor unit de-recruitment during force release (Kamen and De Luca 1989) and an increased reliance on the antagonist to control isometric force release (Spiegel et al. 1996), likely contributes to the differential impact of aging on force release compared to maintenance or generation.

The small sample size in this study limits the ability to precisely estimate effect sizes and to detect small differences between groups. The relationship between performance during a force tracking task and everyday dexterity is unknown, which limits the interpretability of our results. Future studies are planned to examine the mechanisms underlying force release declines in older adults and assess external validity by examining relationships between age, force release characteristics, and a range of gross and fine motor dexterity tasks across a range of younger and older adults.

In conclusion, older adults exhibit a global decline in dynamic force control compared to young adults. This decline is especially pronounced during the force release phase. Possible explanations for these findings include sensory changes, changes in central nervous system circuitry and structure, and peripheral changes in motor units.

## Data Availability

The corresponding author will provide datasets from the current study upon reasonable request.
